# Downregulation of carbonic anhydrase IX expression in mouse xenograft nasopharyngeal carcinoma model via doxorubicin nanobubble combined with ultrasound

**DOI:** 10.1515/med-2024-0910

**Published:** 2024-02-13

**Authors:** Rong Li, Liugui Lu, Zhaoxi Huang, Yong Gao

**Affiliations:** Department of Ultrasound, First Affiliated Hospital of Guangxi Medical University, 530021 Guangxi, China; Department of Ultrasound, First Affiliated Hospital of Guangxi Medical University, 6 Shuangyong Rd, Nanning, 530021 Guangxi, China

**Keywords:** diagnostic ultrasound, nasopharyngeal carcinoma, carbonic anhydrase IX, doxorubicin nanobubbles, microvascular density

## Abstract

The purpose of this study was to investigate whether doxorubicin nanobubbles (DOX-NB) combined with diagnostic ultrasound (DUS) irradiation could downregulate the expression of carbonic anhydrase IX (CAIX) in mouse xenograft nasopharyngeal carcinoma (NPC) model. In this study, the prepared DOX-NB was round and well dispersed. The average diameter of DOX-NB was 250.9 ± 50.8 nm, with an average polydispersity of 0.321 ± 0.05. The cumulative release of DOX in the DOX-NB + DUS group was significantly higher compared with that of the DOX-NB group (*p* < 0.05). DOX-NB combined with DUS irradiation could significantly inhibit cell viability (*p* < 0.05). The expression of CAIX and microvessel density (MVD) in the xenografted tumors was the lowest in the DOX-NB + DUS group compared with that of other groups (*p* < 0.05). In conclusion, DOX-NB combined with DUS irradiation could improve DOX-NB drug release and synergistically inhibit NPC cell activity. DOX-NB combined with DUS irradiation can downregulate the expression of CAIX in mouse xenograft NPC model. This may be due to the synergistic effect of DUS combined with DOX-NB in reducing MVD in NPC.

## 1 Introduction

Nasopharyngeal carcinoma (NPC) has a high prevalence in northern Africa and southeastern Asia, especially Southern China [1]. NPC tends to be more sensitive to ionizing radiation. Therefore, radiotherapy remains the most powerful treatment modality for NPC [2]. However, due to tumor resistance to radiotherapy, certain NPC patients present with local recurrences or distant metastases within 2 years after treatment [3,4]. Thus, tumor resistance to radiotherapy remains a serious obstacle to treatment success in some NPC cases.

Tumor hypoxia is known to be mainly responsible for tumor resistance to radiotherapy or chemotherapy, as well as for promoting tumor phenotypes that result in invasiveness, metastasis, and a poor prognosis [5]. Under the condition of tumor hypoxia, cancer cells become approximately two to three times more radioresistant than they are under normoxic conditions [6]. Hypoxia-induced resistance to radiation remains a serious obstacle in achieving optimal outcomes in patients with NPC [7].

Carbonic anhydrase IX (CAIX) is an important downstream factor of hypoxia inducible factor-1α (HIF-1α), which has a receptor that is mainly expressed in the cell membrane [8]. Some studies have shown that CAIX is associated with tumor radioresistance. High expression of CAIX may indicate poor prognosis [9]. A study of head and neck cancer found that CAIX upregulation correlates with tumor necrosis and microvessel density (MVD) [10]. Regulation of CAIX expression is a new therapeutic target for NPC [11]. However, the methods to block the function of CAIX or regulating its expression are still a challenge [12].

Studies have shown that ultrasound-targeted microbubble destruction (UTMD) technology can increase the permeability of tumor vasculature and effectively enhance the transport and uptake of therapeutic agents [13]. The latest research has shown that microbubbles combined with diagnostic ultrasound (DUS) irradiation could improve the blood perfusion of the tumor [14,15]. At present, no research has been conducted on reducing the expression of CAIX by nanobubbles (NBs) combined with DUS irradiation. In this study, we investigated whether doxorubicin (DOX)-NB combined with DUS irradiation could downregulate the expression of CAIX in NPC, and we preliminarily explored the relationship between its expression level and MVD.

## 2 Materials and methods

### 2.1 Preparation of DOX-NB

The lipids 1,2-dipalmitoyl-sn-glycero-3-phosphocholine, 1,2-dipalmitoyl-sn-glycero-3-phosphate monosodium salt, 1,2-dipalmitoyl-sn-glycero-3-phosphoethanolamine, and mPEG2000-DSPE were dissolved in chloroform in a 4:1:1:1 mass ratio. The solvent was then removed by evaporation, and the lipids were hydrated at 60°C for 30 min in a solution containing 50 μL of glycerol and 1 mL of phosphate-buffered saline (PBS) containing 0.06% Pluronic 124, 0.5% Irgacure 2959, and 0.2% DOX. After hydration, *N,N*′,-diethylaniline and *N*,*N*ʹ-bis(acryloyl)cystamine were dissolved in the lipid solution. The air in the vial was replaced with octafluoropropane (C3F8) using a 10 mL syringe with a needle.

The vial was shaken on an HL-AH series amalgamator (Hangzhou ZhongRun Medical Instrument Co., Ltd) at a high speed for 60 s, then irradiated at 254 nm using an ultraviolet lamp for 30 min. Finally, the mixture was stored upside down at 4°C.

### 2.2 Characterization of DOX-NB

Fluorescence microscopy was used to observe DOX in nanocapsules. The size, zeta potential, and polydispersity index of DOX-NB were measured using a Zetasizer NanoZS instrument (Malvern Instruments, UK).

Bubble size was determined by diluting a sample with PBS at pH 7.4 to a dilution of 1:1,000. Measurements were performed at 25°C with a laser wavelength of 660 nm at an angle of 90°. DOX-NB concentrations were counted using blood cell counting plates. The contrast imaging of DOX-NB *in vitro* was performed in an acrylamide gel phantom with a clinical ultrasound imaging system (LogiQ E9, GE, USA). The DOX-NB samples were diluted with PBS to a dilution of 1:100. Imaging parameters were set to contrast imaging with a frequency of 5.5 MHz, mechanical index (MI) of 0.16, and gain of 5 dB.

### 2.3 Drug loading content (DLC) and encapsulation efficiency

DOX-NB was dialyzed with a dialysis bag (12,000 Da, 5 h) to remove free DOX. The suspension of DOX-NB was freeze-dried for 1 day with a freeze dryer (−55°C, <0.010 mbar). Lyophilized DOX-NB was then weighed and dissolved in PBS. The amount of encapsulated DOX was determined by measuring the absorbance at 483 nm. DOX content is expressed as DLC, which is the ratio of the weight of the loaded DOX to the total weight of the material. Encapsulation efficiency (%EE) is the percentage of encapsulated DOX to initial feeding DOX.

### 2.4 DOX release from DOX-NB *in vitro*

DOX-NB was enclosed in a dialysis bag (cutoff: 12,000 Da), placed in a container with 100 mL of PBS, and shaken at 100 rpm at a temperature of 37°C. The DOX-NB + DUS group was irradiated by DUS (LogiQ E9, GE, USA; probe frequency: 5.5 MHz, MI: 1.2, thermal index in soft tissue [TIS]: 0.3) for 3 min. DOX release was measured for up to 24 h, withdrawing 1 mL at each fixed time and replacing it with 1 mL of PBS. The concentration of DOX in the external buffer was measured using a Synergy H1 microplate reader at 483 nm. The experiments were performed in triplicate.

### 2.5 Cell culture

NPC cells (5-8F) were purchased from Shanghai Yaji Biotech Co., Ltd. The cells were cultured in 1640 complete medium (10% fetal bovine serum, 1% penicillin–streptomycin) and placed in a humidified atmosphere (37°C, 5% CO_2_). Cells in the logarithmic growth phase were harvested for the experiments.

### 2.6 Effects of DOX-NB on 5-8F cells *in vitro*

The viability of the 5-8F cells was measured using a Cell Counting Kit-8 (CCK-8). The cells were cultured in 1640 complete medium (10% fetal bovine serum, 1% penicillin–streptomycin) and placed in a humidified atmosphere (37°C, 5% CO_2_). Cells were passaged until they were 90% confluent and then detached with 0.25% trypsin-EDTA. The 5-8F cells (at 8 × 10^3^ cells per plate) were seeded into 96-well plates. After 24 h of culture, the surface medium was aspirated and the cells were randomly divided into five groups: Control, DOX, DOX + DUS, DOX-NB, and DOX-NB + DUS. The DUS parameters were set as follows: frequency, 5.5 MHz; MI, 1.2; TIS, 0.3; and irradiation time, 3 min. After 3 h of incubation at 37°C, the treatment solution was removed. The cells were washed with PBS three times. Then, 10 μL of CCK-8 solution was added to each well, and the cells were cultured for 1.5 h. A microplate reader (Synergy H1, BioTek Instruments, USA) was used to determine the absorbance at 450 nm.

### 2.7 Apoptosis experiment

Annexin V-FITC and propyridine iodide (PI) apoptosis kit (MultiSciences Biotech Co., Ltd) were used to detect apoptotic cells. The 5-8F cells (at 6 × 10^5^ cells per plate) were seeded into 35 mm cell culture dishes. After 24 h of culture, the cells were randomly divided into five groups: Control, DOX, DOX + DUS, DOX-NB, and DOX-NB + DUS. The DUS parameters were set as follows: frequency, 5.5 MHz; MI, 1.2; TIS, 0.3; and irradiation time, 3 min. After 3 h of incubation at 37°C, the treatment solution was removed. The cells were washed with PBS. Then, complete medium was added to each dish and the cells were cultured in a humidified atmosphere (37°C, 5% CO_2_). After 24 h of culture, 5-8F cells were collected and washed with PBS two times. The cells were resuspended in 500 µL 1× binding buffer, stained with 5 µL Annexin V-FITC and 10 µL PI for 5 min at room temperature in the dark. Apoptosis was analyzed with a BD FACS flow cytometer (BD Bioscience, USA).

### 2.8 Animal preparation and subcutaneous implantation models

The animals were handled according to the Institutional Animal Care and Use Committee. The study was approved by the Ethics Committee of Guangxi Medical University (Approval number 202109016). BALB/c-nu nude mice (4–6 weeks old) were purchased from Guangxi Medical University Lab Animal Research Center. The 5-8F cells and 200 µL of Matrigel mixture (1 × 10^6^ cells/mL) were injected subcutaneously into the hind flanks of the mice. When the tumor diameter reached 8–10 mm, the mice were randomly divided into five groups (five mice/group): Control, DOX, DOX + DUS, DOX-NB, and DOX-NB + DUS. The mice were then anesthetized with 3% pentobarbital sodium (0.1 mL/10 g). Each mouse was injected once with 200 μL sample solution via tail vein injection. The DOX intake of all treatment groups was 184 nmol (5 mg/kg). One minute after the injection of the sample solution via the tail vein, the mice in the DOX + DUS group or DOX-NB + DUS group received ultrasound irradiation on the tumor surface, using DUS instruments (frequency: 5.5 MHz, MI: 1.2, TIS: 0.3, and irradiation time: 3 min). After 3 h, the mice were sacrificed by euthanasia and by CO_2_ asphyxiation. Tumors were collected for analysis.

### 2.9 Detection of CAIX expression in tumor tissues by immunohistochemistry

The tumors extracted from each group were fixed with 4% paraformaldehyde for 24 h (4°C) and then embedded in paraffin. The tissues were sectioned after dewaxing (thickness 4 µm), and then immunohistochemical CAIX detection was performed. The presence of brown granules on the cytomembrane was considered to be the positive expression of CAIX. Five high-magnification fields (×400) were randomly selected from each section under a light microscope. The representative figures were captured and semiquantitatively analyzed using ImageJ. The integrated optical density (IOD) was measured as the expression of CAIX in the target area, and the average IOD of five fields was calculated for the final analysis.

### 2.10 Quantification of MVD in xenograft tumors by immunohistochemistry

Paraffin-embedded blocks were retrieved and cut into 4 µm sections. All sections underwent dewaxing, rehydration, and heat-mediated antigen retrieval with Tris-EDTA buffer (pH 9.0, epitope retrieval solution) for 20 min. They were then subjected to endogenous peroxidase blocking. After cooling at room temperature, they were rinsed in PBS and incubated overnight at 4°C with rabbit anti-CD31 monoclonal antibody. Next, the slides were incubated with a goat anti-rabbit antibody for 45 min at 37°C. Five high-magnification fields (200×) were selected randomly from each section under a light microscope to count the number of microvessels and determine the MVD with Image J software.

### 2.11 Statistical analysis

All experiments were performed in triplicate, and the data were presented as mean ± standard deviation. Statistical analyses were performed using SPSS version 25.0. Differences between the two groups were analyzed using the two-way repeated measure analysis of variance (ANOVA). Differences among multiple groups were evaluated using one-way ANOVA). A *p*-value <0.05 was considered to be statistically significant.

## 3 Results

### 3.1 DOX-NB characterization

**Figure 1:**
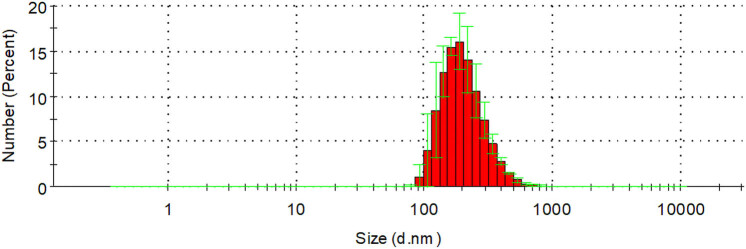
Size distribution of the DOX-NB obtained by Zetasizer NanoZS.

**Figure 2:**
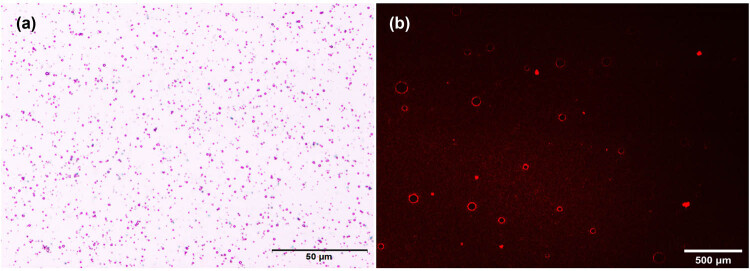
(a) DOX-NB under an optical microscope (×400). (b) Fluorescence microscope image of DOX-NB (×400) (scale bar = 500 μm).

**Figure 3:**
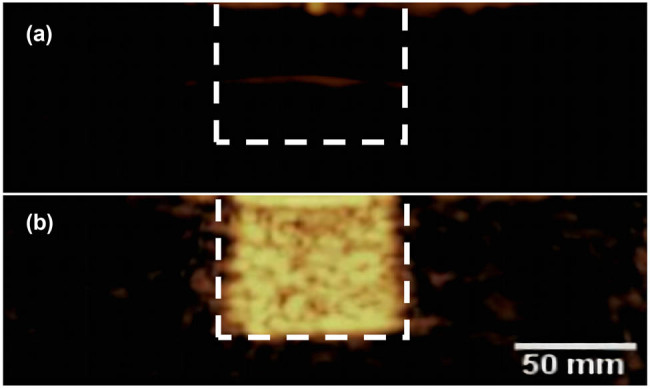
(a) Contrast-enhanced ultrasound image of PBS buffer shows an anechoic dark area. (b) DOX-NB contrast-enhanced ultrasound image shows a dense and uniform high enhanced area.

The average diameter of DOX-NB was 250.9 ± 50.8 nm, with an average polydispersity of 0.321 ± 0.05 (Figure 1). The zeta potential was −23.6 ± 2.21 mV measured by the particle size and zeta potential analyzer. The DOX-NBs were round and well dispersed, with a density of 9.28 × 10^8^/mL under an optical microscope (Figure 2a). A fluorescence microscope was used to clearly show the red fluorescence signals of DOX on the shell of DOX-NB (Figure 2b). Figure 3a shows the contrast-enhanced ultrasound image of PBS buffer as an anechoic dark area, while Figure 3b shows DOX-NB as a strong and uniform high enhancement area.

### 3.2 Loading and DOX release from DOX-NB *in vitro*

**Figure 4:**
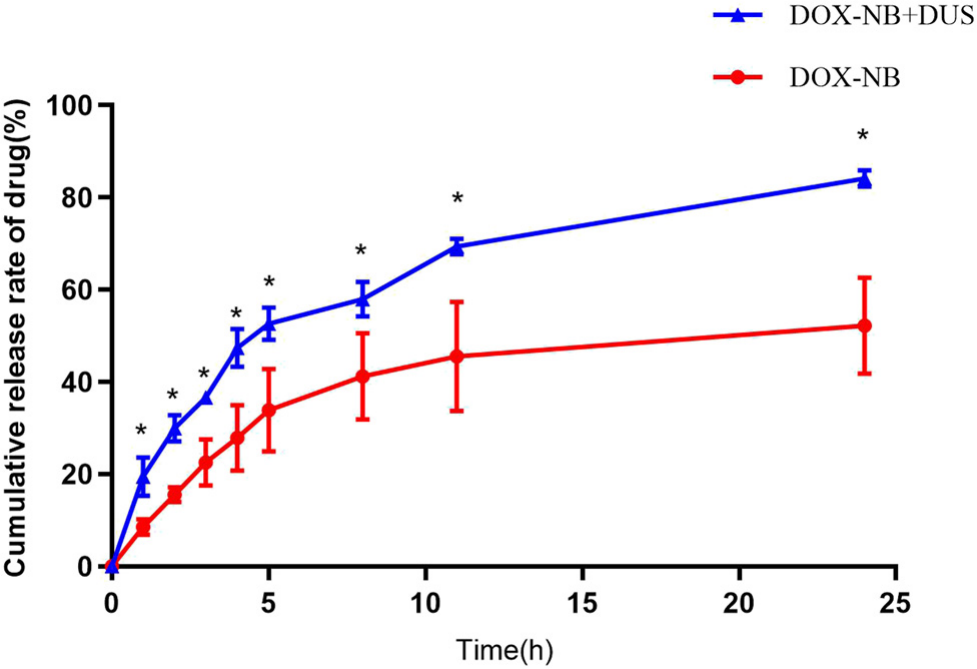
DOX release curve of DOX-NB + DUS group compared with DOX-NB group within 24 h *in vitro* (*n* = 3/group).

Encapsulated DOX content in particles was determined using the dialysis technique. The DLC of DOX-NB was 10.17 ± 4.79 μg/mg. The encapsulation efficiency was 23.4 ± 0.08%. Figure 4 shows the release curve of DOX from DOX-NB with and without DUS irradiation *in vitro*. The cumulative of DOX released from DOX-NB was significantly different between the DOX-NB + DUS group and the DOX-NB group (*p <* 0.05) (Table A1). The DOX-NB group released 52.2% of the DOX after 24 h. However, 84.1% of the DOX was released in the DOX-NB + DUS group.

### 3.3 DOX cytotoxicity *in vitro*

**Figure 5:**
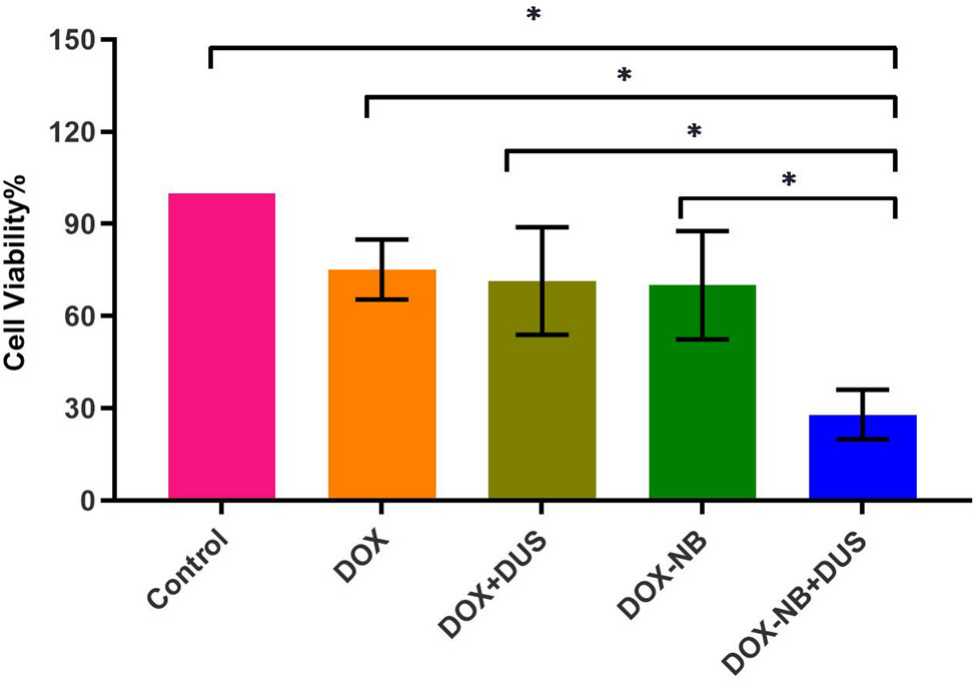
Cell viability of 5-8F cells (*n* = 3/group) with different treatments (*p < 0.05).

To investigate the anticancer effects *in vitro*, the viability of 5-8F cells was measured using a CCK-8 assay. The cell viability results were 27.7 ± 8.08% in the DOX-NB + DUS group, 67.6 ± 15.99% in the DOX-NB group, 71.1 ± 17.50% in the DOX + DUS group, 77.8 ± 5.85% in the DOX group, and 100% in the Control group (Figure 5, Table A2). The viability of 5-8F cells in the DOX-NB + DUS group was lower than that in the other groups (*p* < 0.05), suggesting that DOX-NB combined with ultrasound irradiation had a better inhibitory effect on the viability of 5-8F cells.

**Figure 6:**
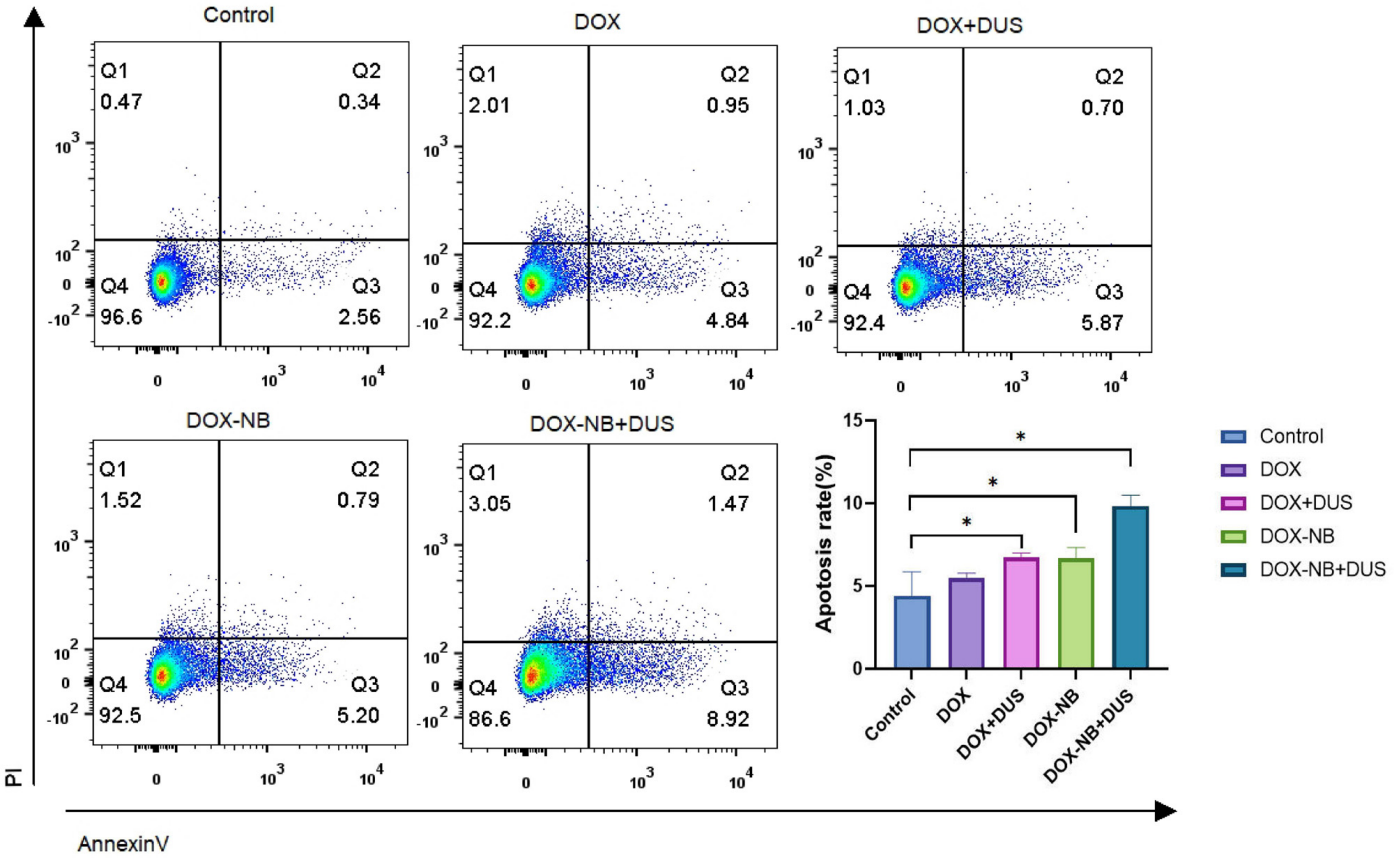
Apoptosis rate of 5-8F cells (*n* = 3/group) with different treatments (*p < 0.05).

To further investigate the anticancer effects *in vitro*, we used flow cytometry to assess the apoptosis of 5-8F cells. Annexin V-FITC/PI apoptosis kit was used to detect the induction of apoptosis. The apoptosis rate of cells was 9.8 ± 0.52% in the DOX-NB + DUS group, 6.7 ± 0.52% in the DOX-NB group, 6.7 ± 0.21% in the DOX + DUS group, 5.5 ± 0.24% in the DOX group, and 4.4 ± 1.18% in the Control group (Figure 6, Table A3). The apoptosis rate of 5-8F cells in the DOX-NB + DUS group was higher than that in the other groups (*p* < 0.05), further verifying that DOX-NB combined with ultrasound irradiation inhibited the viability of 5-8F cells well.

### 3.4 Expression of CAIX antigen by immunohistochemistry *in vivo*

**Figure 7:**
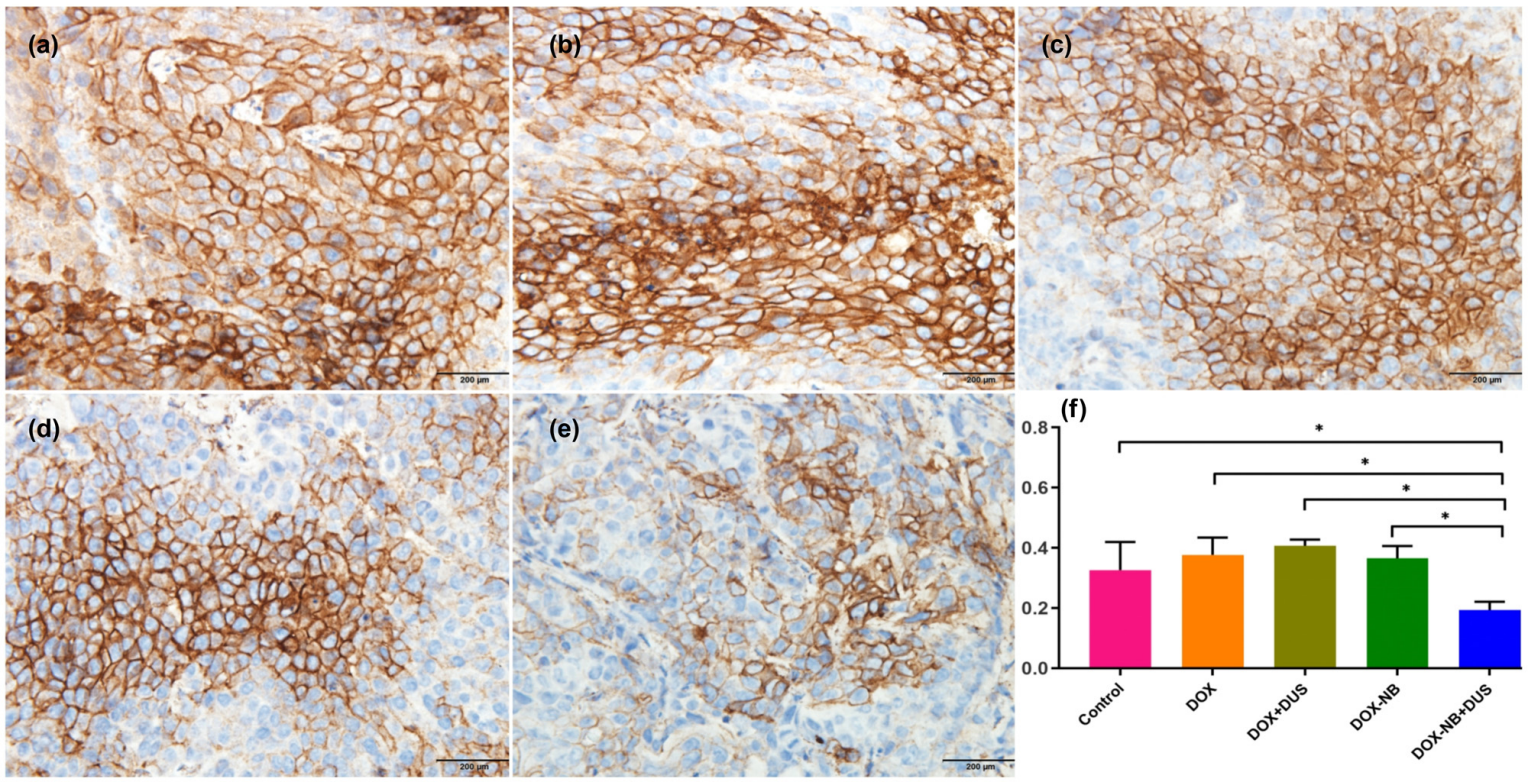
Different expressions of CAIX by immunohistochemical staining in xenografted tumors in different treatment groups (*n* = 5/group). (a–e) Representative immunohistochemical staining of CAIX: (a) Control group, (b) DOX group, (c) DOX + DUS group, (d) DOX-NB group, (e) DOX-NB + DUS group (×400) (scale bar = 200 μm). (f) Quantification of CAIX as presented in (a)–(e) (*p < 0.05).

Figure 7 exhibits immunohistochemical images of the CAIX-stained in xenografted tumor. The expression of CAIX in the xenografted tumors was downregulated in the DOX-NB + DUS group compared with that in the other groups (*p* < 0.05) (Table A4).

### 3.5 Expression of MVD in tumor tissues by immunohistochemistry

**Figure 8:**
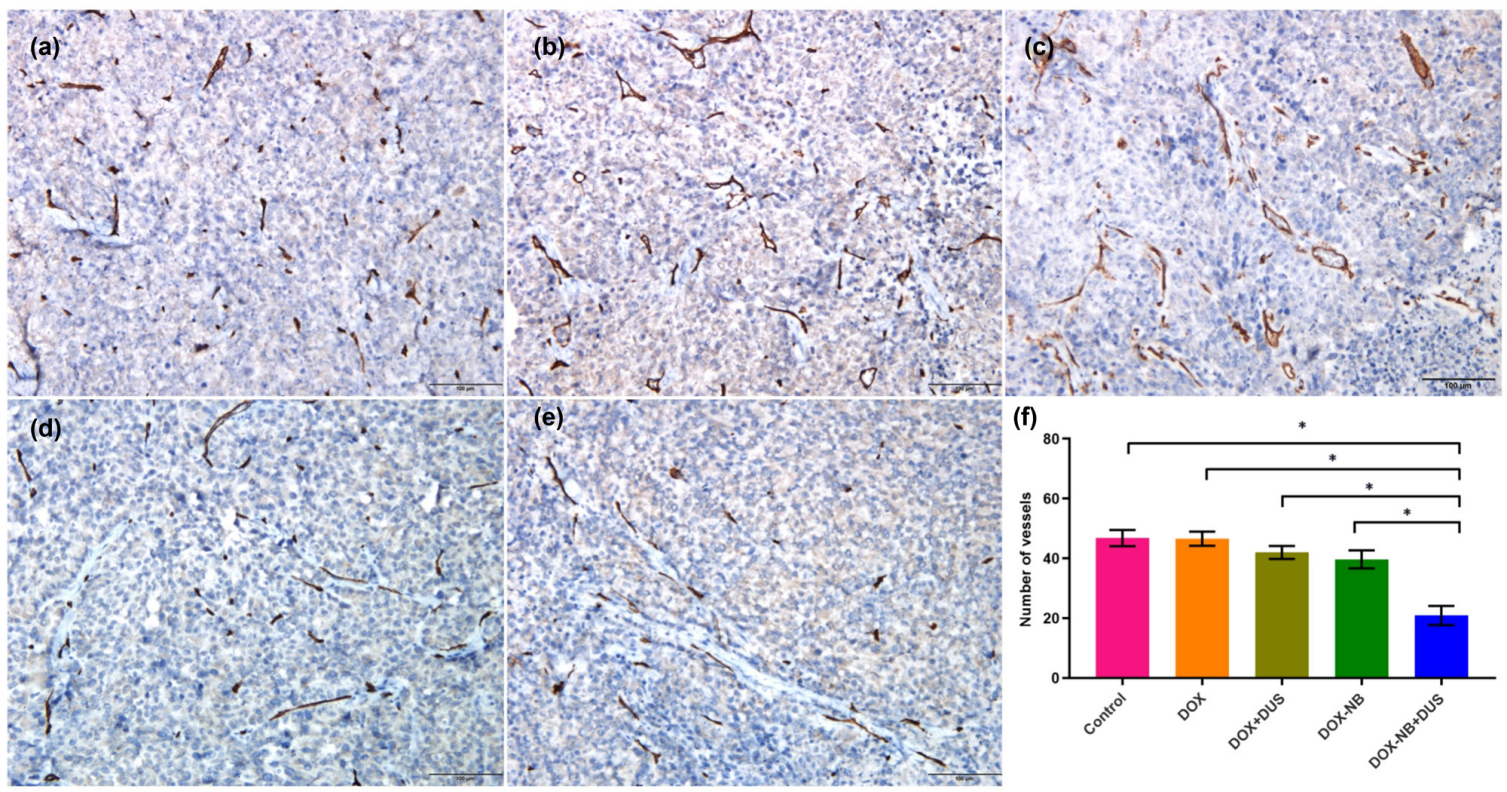
Different expressions of CD31 by immunohistochemical staining in xenografted tumors in different treatment groups (*n* = 5/group). (a–e) Representative immunohistochemical staining of CD31: (a) Control group, (b) DOX group, (c) DOX + DUS group, (d) DOX-NB group, (e) DOX-NB + DUS group (×200) (scale bar = 100 μm). (f) Quantification of CD31 as presented in (a)–(e) (*p < 0.05).

To determine the effects of treatment on tumor angiogenesis, immunohistochemical staining of CD31 was performed to quantify MVD in xenograft tumors (Figure 8). The MVDs of the DOX-NB + DUS, DOX-NB, DOX + DUS, DOX, and Control groups were calculated as 20.90 ± 3.18, 39.70 ± 2.98, 42.00 ± 2.16, 46.60 ± 2.37, and 46.80 ± 2.70, respectively. The difference between the Control group and DOX groups was not significant (*p* = 0.869). Compared with the DOX + DUS group, the MVD of the DOX-NB group was decreased, but the difference was not statistically significant (*p* = 0.064). The DOX-NB + DUS group had the lowest MVD compared with the other groups (*p* < 0.05) (Table A5).

## 4 Discussion

Tumor hypoxia has been demonstrated in 100% of primary NPC and closely related to tumor radiotherapy resistance [16]. Therefore, hypoxia is increasingly being recognized as an important therapeutic target to improve tumor radiosensitivity. CAIX expression has been demonstrated to correlate with tumor hypoxia [17]. Jiang et al. showed that downregulation of CAIX expression could promote radiosensitivity in CNE-2 cells [18].

In this study, the expression of CAIX in xenografted tumors of NPC was significantly downregulated when treated with DOX-NB + DUS group compared with DOX + DUS group or DOX-NB group, as determined using immunohistochemical staining. A previous study found that the expression of CAIX in head and neck cancer cells is positively correlated with MVD [19]. Reijnen et al. found that the poor outcome in hypoxic endometrial carcinoma may be related to vascular density and the expression of CAIX [20]. Rubio-Briones et al. found that the expressions of MVD and the CAIX were related to histological subtypes. The expression of CAIX can be used as a surrogate indicator of microvascular density [21]. To prove this relationship, the present study further detected the expression of MVD labeled by CD31 in subcutaneous transplanted tumors of NPC with different treatment groups. The results indicated that MVD was significantly lower when treated with DOX-NB + DUS compared with other treatment groups. Therefore, DOX-NB combined with DUS irradiation can downregulate the expression of CAIX in xenograft tumor, which is possibly related to the decrease in MVD within the tumor.

The reason for the decrease in MVD may be related to the cavitation effect induced by UTMD. Studies have shown that with appropriate ultrasound pressure, this cavitation effect can produce microfluidics, which can cause endothelial cell damage and microvascular rupture, thereby achieving the effect of destroying tumor microvessels [22]. Another reason for the decrease in MVD may be related to the increasing DOX concentration within the tumor. *In vitro*, we found that DOX-NB could effectively release DOX after exposure to DUS irradiation. In cell viability experiments, the viability of 5-8F cells in the DOX-NB + DUS group was significantly reduced compared with the other treatment groups (Control, DOX, DOX + DUS, and DOX-NB). The apoptosis rate of 5-8F cells in the DOX-NB + DUS group was significantly higher compared with the other treatment groups (Control, DOX, DOX + DUS, and DOX-NB). It was indicated that DOX-NB combined with DUS irradiation could improve the intracellular uptake of DOX *in vitro*. On the one hand, UTMD could produce instantaneous pores on the cell membrane and increase its permeability, which could lead to more DOX entering the tumor cells [13]. On the other hand, UTMD could promote the release of DOX encapsulated in drug-loaded nanovesicles and increase the concentration of DOX in tumor cells. It can effectively enhance the transport and uptake of DOX [23,24]. Studies have shown that chemotherapy not only kills tumor cells but also destroys the blood supply of tumor tissue by killing or inhibiting activated endothelial cells on blood vessels.

Research has illustrated that vascular endothelial cells are 10–10,000 times more sensitive to cytotoxic drugs than tumor cells are. Most tumor chemotherapy drugs have the effect of blocking angiogenesis and destroying existing blood vessels, including DOX [25]. Interestingly, this study showed that there was no statistically significant difference in MVD between the free DOX group and the Control group. Such a difference was also lacking between the DOX + DUS group and the DOX-NB group. This indicated that without the synergistic effect of UTMD, the low dose of DOX could not effectively exert its anti-tumor angiogenesis effect under the lower DOX concentration used in this study. The increasing concentration of DOX in the tumor could not only enhance the anti-tumor effect but also increase the ability to inhibit angiogenesis.

This study had a few limitations. First, in cell experiments, the mechanism of UTMD on cells has not been thoroughly studied, such as using flow cytometry to detect the effect of UTMD on cell cycle and using electron microscopy to observe the effect of cavitation on cell structure. Second, we have not constructed a cell hypoxia model to evaluate the changes of CAIX in cells after treatment. Third, we have not performed survival experiments to observe the inhibitory effect of treatment on tumor size.

## 5 Conclusion

DOX-NB combined with DUS irradiation could improve DOX-NB drug release and synergistically inhibit NPC cell activity. Meanwhile, DOX-NB combined with DUS irradiation could downregulate the expression of CAIX and decrease the MVD in mouse xenograft NPC model. This may be due to the synergistic effect of DUS combined with DOX-NB in reducing MVD in the context of NPC. Therefore, DOX-NB combined with DUS irradiation may be a promising therapeutic method for NPC.

## Appendix

**Table A1:** The DOX release rate of DOX-NB + DUS group compared with DOX-NB group within 24 h in vitro(n = 3/group)

Time (h)	Comulative release rate of DOX (%)		*P*-value
	DOX-NB + DUS	DOX-NB	
1	19.5 ± 4.19	8.6 ± 1.70	0.014*
2	29.9 ± 2.84	15.5 ± 1.61	0.002*
3	36.6 ± 1.35	22.5 ± 5.02	0.009*
4	47.3 ± 4.16	27.8 ± 7.15	0.015*
5	52.6 ± 3.54	33.9 ± 8.917	0.028*
8	57.9 ± 3.81	41.2 ± 9.35	0.046*
11	69.3 ± 1.72	45.5 ± 11.85	0.026*
24	84.1 ± 1.78	52.2 ± 10.42	0.006*

**p* < 0.05.

**Table A2:** The Cell viability of 5–8 F cells with different treatments (n = 3/group)

Group	Group	*P*-value
Control	DOX	< 0.001*
	DOX + DUS	0.026*
	DOX-NB	0.009*
	DOX-NB + DUS	< 0.001*
DOX	DOX + DUS	0.969
	DOX-NB	0.704
	DOX-NB + DUS	< 0.001*
DOX + DUS	DOX-NB	1.000
	DOX-NB + DUS	0.002*
DOX-NB	DOX-NB + DUS	0.001*

**p* < 0.05.

**Table A3:** The apoptosis rate of 5–8 F cells with different treatments (*n* = 3/group)

Group	Group	*P*-value
Control	DOX	1.26
	DOX + DUS	0.005*
	DOX-NB	0.005*
	DOX-NB + DUS	< 0.001*
DOX	DOX + DUS	0.079
	DOX-NB	0.089
	DOX-NB + DUS	< 0.001*
DOX + DUS	DOX-NB	0.943
	DOX-NB + DUS	0.001*
DOX-NB	DOX-NB + DUS	0.001*

**p* < 0.05.

**Table A4:** The expression of CAIX by Immunohistochemistry in differrence treatment group(n = 5/group)

Group	Group	*P*-value
Control	DOX	0.001*
	DOX + DUS	< 0.001*
	DOX-NB	< 0.001*
	DOX-NB + DUS	< 0.001*
DOX	DOX + DUS	0.277
	DOX-NB	0.102
	DOX-NB + DUS	< 0.001*
DOX + DUS	DOX-NB	0.570
	DOX-NB + DUS	< 0.001*
DOX-NB	DOX-NB + DUS	< 0.001*

**p* < 0.05.

**Table A5:** The expression of CD31 by Immunohistochemistry in differrence treatment group(n = 5/group)

Group	Group	*P*-value
Control	DOX	0.869
	DOX + DUS	< 0.001*
	DOX-NB	< 0.001*
	DOX-NB + DUS	< 0.001*
DOX	DOX + DUS	< 0.001*
	DOX-NB	< 0.001*
	DOX-NB + DUS	< 0.001*
DOX + DUS	DOX-NB	0.064
	DOX-NB + DUS	< 0.001*
DOX-NB	DOX-NB + DUS	< 0.001*

**p* < 0.05.
